# The VicGeneration study - a birth cohort to examine the environmental, behavioural and biological predictors of early childhood caries: background, aims and methods

**DOI:** 10.1186/1471-2458-10-97

**Published:** 2010-02-25

**Authors:** Andrea M de Silva-Sanigorski, Hanny Calache, Mark Gussy, Stuart Dashper, Jane Gibson, Elizabeth Waters

**Affiliations:** 1 Melbourne School of Population Health, University of Melbourne, Carlton, Australia; 2 WHO Collaborating Centre for Obesity Prevention, Deakin University, Geelong, Australia; 3 Dental Health Services Victoria, Carlton, Australia; 4 School of Dentistry and Oral Health, LaTrobe University, Bendigo, Australia; 5 Oral Health CRC, University of Melbourne, Carlton, Australia

## Abstract

**Background:**

Dental caries (decay) during childhood is largely preventable however it remains a significant and costly public health concern, identified as the most prevalent chronic disease of childhood. Caries in children aged less than five years (early childhood caries) is a rapid and progressive disease that can be painful and debilitating, and significantly increases the likelihood of poor child growth, development and social outcomes. Early childhood caries may also result in a substantial social burden on families and significant costs to the public health system. A disproportionate burden of disease is also experienced by disadvantaged populations.

**Methods/Design:**

This study involves the establishment of a birth cohort in disadvantaged communities in Victoria, Australia. Children will be followed for at least 18 months and the data gathered will explore longitudinal relationships and generate new evidence on the natural history of early childhood caries, the prevalence of the disease and relative contributions of risk and protective biological, environmental and behavioural factors. Specifically, the study aims to:

1. Describe the natural history of early childhood caries (at ages 1, 6, 12 and 18 months), tracking pathways from early bacterial colonisation, through non-cavitated enamel white spot lesions to cavitated lesions extending into dentine.

2. Enumerate oral bacterial species in the saliva of infants and their primary care giver.

3. Identify the strength of concurrent associations between early childhood caries and putative risk and protective factors, including biological (eg microbiota, saliva), environmental (fluoride exposure) and socio-behavioural factors (proximal factors such as: feeding practices and oral hygiene; and distal factors such as parental health behaviours, physical health, coping and broader socio-economic conditions).

4. Quantify the longitudinal relationships between these factors and the development and progression of early childhood caries from age 1-18 months.

**Discussion:**

There is currently a lack of research describing the natural history of early childhood caries in very young children, or exploring the interactions between risk and protective factors that extend to include contemporary measures of socio-behavioural factors. This study will generate knowledge about pathways, prevalence and preventive opportunities for early childhood caries, the most prevalent child health inequality.

## Background

Dental caries (decay) during childhood is largely preventable however it remains a significant and costly public health concern, identified as the most prevalent chronic disease of childhood [1]. Caries in children aged less than five years (Early Childhood Caries; ECC) is a rapid and progressive disease that can be painful and debilitating, and significantly increases the likelihood of poor child growth, development and social outcomes. The short-term sequelae of untreated ECC are pain, infection and abscesses [2] and by 6 years of age approximately 40% of Australian children have some dental decay, of which 60% goes untreated [3]. ECC has wide-ranging health and developmental consequences, including delayed growth, reduced general physical health, nutritional and sleep problems and the potential for disrupted social and academic development due to school absences [4,5]. Caries in young children is also difficult and expensive to manage and antibiotics, general anaesthesia and hospital admission may be required for treatment [6].

Knowledge regarding the prevalence of dental caries and its treatment in early childhood is currently limited in Australia, as in many countries [7], due commonly to the lack of coordinated, funded monitoring systems, and difficulties faced in accessing this population group [8,9]. A review of the literature suggests that in most developed countries the prevalence rate of ECC is between 1% and 12%[7]. However in less developed countries and in disadvantaged groups within developed countries the prevalence has been reported to be as high as 70% [5].

Consistent with the universal social gradient that exists across areas of general health and wellbeing, caries rates are higher among the more socially disadvantaged [10-12], particularly in early childhood, and particularly for children who are refugees or migrants, or whose parents are refugees or migrants from a non-English speaking background [13]. This can arise from socioeconomic disadvantage, social exclusion and socio-cultural differences in oral health beliefs and practices [14-16].

While much is known about the causes of dental caries in general, the development of caries in very early life is less well understood. Caries is a diet related, infectious and transmissible disease [17] of multi-factorial aetiology [18-20], and the important protective role of exposure to fluoride is well-established [21]. However, in early childhood, routes of transmission, the timing of bacterial colonization and the relative composition of the bacterial species involved in caries development remain unclear. Additionally, parents (rather than the child) are the key individuals who determine the social and behavioural environment that shapes oral health practices.

To date, investigations of the development of ECC have almost exclusively focussed on either biological factors or socio-environmental factors, and studies that combine measurement of both areas are rare. While family and broader socio-environmental influences on oral health have become an increasing focus of attention [16,22,23], little research has examined how these factors interact with pathobiological and exposure factors to determine the onset and progression of caries in young children. This study will employ a longitudinal, multi-disciplinary design to investigate the factors involved in the development of ECC, providing an evidence-base to inform interventions that target ECC risk and protective factors.

Specifically this study aims to:

1. Describe the natural history of ECC (at ages 1, 6, 12 and 18 months), tracking pathways from early bacterial colonisation, through non-cavitated enamel white spot lesions to cavitated lesions extending into dentine.

2. Determine the oral bacterial species and their relative abundance in the saliva of infants and their primary care giver.

3. Identify the strength of concurrent associations between ECC and putative risk and protective factors, including biological (microflora, saliva, tooth susceptibility), environmental (fluoride exposure) and socio-behavioural factors (proximal factors such as: feeding practices and oral hygiene; distal factors such as: parental health behaviours, physical health, stress and coping and broader socio-economic conditions).

4. Quantify the longitudinal relationships between these factors and the development and progression of ECC from age 1-18 months.

The specific hypotheses to be tested are:

1. Higher levels of Mutans Streptococci (MS) in saliva, lower salivary buffering capacity and lower levels of salivary fluoride, calcium and phosphate are associated concurrently with higher rates of white spot lesions, cavitation (decayed), missing and filled teeth at ages 6, 12 and 18 months.

2. After accounting for levels of child MS and salivary characteristics: Higher levels of maternal MS and lower infant exposure to fluoride (water or toothpaste) are associated concurrently with higher rates of white spot lesions, cavitation (decayed), missing and filled teeth at ages 6, 12 and 18 months.

3. After accounting for levels of child MS and salivary characteristics: adverse parental health behaviours (eg consumption of sweetened beverages, poor oral self-care, smoking), lack of support for infant oral health (infrequent tooth-brushing, non-fluoride toothpaste) and adverse feeding practices (consumption of sweetened beverages, shared utensils) will be associated concurrently with higher rates of white spot lesions, cavitation (decayed), missing and filled teeth at ages 6, 12 and 18 months.

4. Higher levels of child MS, lower salivary buffering capacity and lower levels of salivary fluoride, calcium and phosphate (at ages 1, 6, 12, and 18 months) will be associated prospectively with higher rates of white spot lesions, cavitation (decayed), missing and filled teeth at ages 6, 12 and 18 months.

5. After accounting for concurrent biological and exposure factors: adverse parental health behaviours, lack of support for infant oral health and adverse feeding practices (at ages 1, 6, 12 and 18 months) will be associated prospectively with higher rates of white spot lesions, cavitation (decayed), missing and filled teeth at ages 6, 12 and 18 months.

6. Finally, because socio-ecological risk factors typically operate in an interactive manner (such that the effect of additional risks is more than additive [24]), after accounting for concurrent risk and protective factors, the accumulation of risk factors across previous measurement periods, will be associated with an exponential increase in rates of white spot lesions, cavitation (decayed), missing and filled teeth at each age.

## Methods/Design

**Figure 1:**
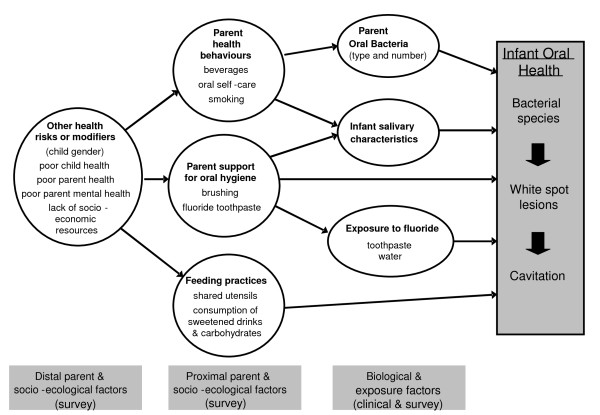
**The proposed direct and indirect pathways related to the development of early childhood caries**.

This research will employ a longitudinal, multi-disciplinary design to investigate the factors involved in the development of ECC within the socio-ecological model of health (see figure 1).

### Sampling design and recruitment

**Figure 2:**
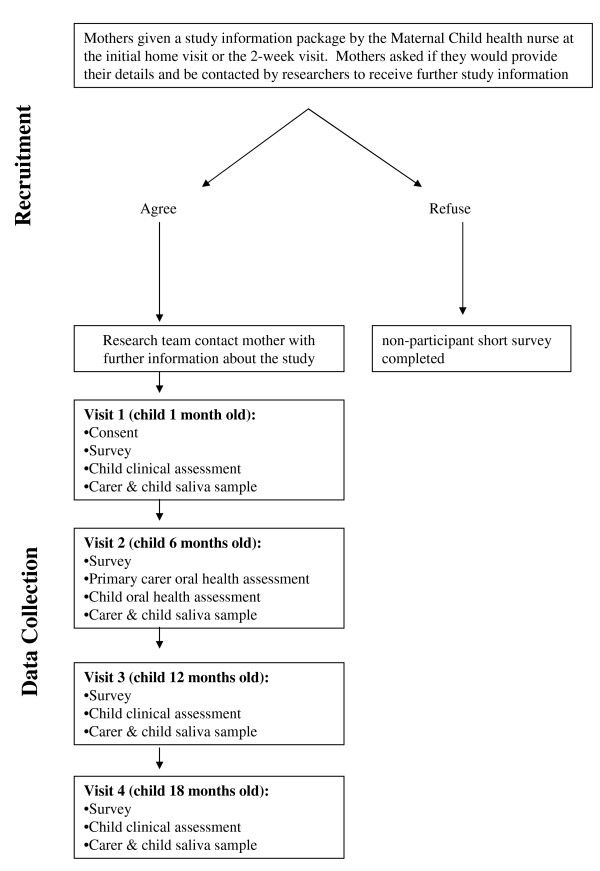
**Flow diagram of the recruitment and data collection procedures for the VicGeneration study**.

The study will involve the establishment of a birth cohort of 450 infants and their primary carers, assessed at four time points: ages 1, 6, 12 and 18 months (see figure 2). The parents of children born in 2009 from select rural, regional and metropolitan Local Government Areas (LGAs) in Victoria, Australia will be invited to participate. The selection of the LGAs will be based on the level of disadvantage, cultural diversity, birth rate, and acceptability and feasibility of data collection. The metropolitan LGAs will provide a culturally and linguistically diverse (CALD) sample, and all LGAs will have a high representation of families that are socially or economically disadvantaged. The LGAs will also be those not receiving specific community-wide health promotion interventions. Prior to recruitment, researchers will consult with the local government Maternal and Child Health (MCH) service managers, the MCH nurses and community dental service providers.

#### Recruitment

Newborns and their parents will be recruited several weeks after birth via the MCH service. The MCH service is the primary point of contact for children aged 0 to 4 years and their carers/parents, with a total of 6 scheduled universal child health checks in the first 12 months (at ages 2 weeks, 4 weeks, 8 weeks, 4 months, 8 months and 12 months)[8]. For recruitment, MCH Nurses (MCHNs) will provide a study information package to all eligible mothers who attend for their child's 2 or 4 week child health check, providing (via phone, fax or email) the names and phone numbers of any mothers who agree to be contacted by the research team for further study information. These prospective participants will then be contacted by the researchers (by phone) to explain the research study, invite participation and schedule the first (Time 1) assessment.

##### Exclusion criteria

From clinic records and during service provision MCHNs will identify and exclude families with the following characteristics: those who intend to move location within the next 12 months; the child requires specialist paediatric care; there is severe illness in the family; or the presence of parental mental illness. These factors are deemed to potentially limit research participation or present an unreasonable additional burden on the family. Descriptive non-identifying data will be collected by MCHNs about non-participant families (excluded and refusals) to enable analyses of non-participation biases.

### Sample Size

For the overall birth cohort we estimate a logistically feasible maximum with respect to fieldwork and resource allocation to be 450 infants and their parents [25]. Therefore, the final sample will be the first 450 families to provide consent and Time 1 (baseline) data. From our previous experience we predict the overall prevalence of ECC (frank cavitation and incipient or white spot lesions) to be ~8% [25], therefore a final sample of 400 would provide sufficient power for testing the study's primary aims. Specifically, we expect our overall prevalence estimates to be able to be quoted with 95% confidence intervals of the order of ± 2.6% (Aims 1 & 2). For any group comparisons undertaken we would be able to detect absolute differences of 9% or greater in ECC prevalence between groups of equal sizes (e.g. expected for the rurality variable) with 80% power. Finally, for Aims 3 & 4, our analyses would consider a path analytic approach to permit the modelling of the complex relationships between variables and across time as defined by the socio-ecological model of the development of ECC.

### Bacteriology nested case-control study

Within the larger study we will nest a case-control study to test the hypothesis that increased number of MS detected in the saliva, lower salivary buffering capacity and lower levels of salivary fluoride, calcium and phosphate are associated with the risk of ECC. Cases will be children with clinical diagnosis of ECC plus their caregivers. Controls will be clinically diagnosed ECC-free children plus their caregivers. Based on epidemiological trajectory data of rural populations [15,25] we anticipate that approximately 8% of the 400 infants will develop ECC. We will analyse and compare the salivary samples of those 8% (n = 32) plus their caregivers (n = 32) with healthy controls (ECC free children plus their caregivers; n = 64). The sample size accounts for an attrition rate of about 10%. Controls will be randomly selected from our caries-free study population using statistical software. It is established in the literature that majority of individuals with active caries show high levels of MS [17] and assuming that in our population a large number (up to 80%) of ECC cases demonstrate presence of MS, we shall be able to detect absolute differences in MS prevalence of 25% or greater between ECC cases and controls with 80% power.

### Measures

Measurement tools comprise previously validated methods, scales and items to collect survey and clinical data. Saliva samples will be collected from the mother (or primary care giver) and child, oral health professionals will conduct assessments of maternal and child oral health status, and parent-completed questionnaires will be used to capture information on oral health related behaviours, attitudes, knowledge, skills and practices, as well as family socio-demographics (repeated at each data collection point). Parents will be offered the choice of a home visit or attendance at their MCH centre for data collection. Clinical examinations and saliva collection will take approximately 30 minutes to complete and the parent-reported questionnaire will also take approximately 30 minutes to complete. In a study such as this, it is necessary to employ brief items and scales that are reliable and valid indicators of the variables of interest, without over-burdening participants. Where possible, the measures selected have been drawn from the Longitudinal Study of Australian Children (LSAC)[26] and population health surveys previously used in Victoria.

### Primary outcomes

#### Dental caries

This will be assessed by dental examination, principally as non-cavitated white spot lesions, and also recorded as decayed, missing and filled teeth using the Modified ICDAS II method (see below). Non-cavitated white spot lesions are pre-cavitated lesions that are the clinically visible (opaque, white) first signs of caries. White spot lesions can re-mineralise and not progress to cavitation.

#### Parent-reported measures

The parent questionnaire will enable an assessment of sample characteristics, and potential behavioural and environmental risk and protective factors for ECC.

#### Proximal variables

*Maternal health and health behaviours: *will include items to assess a range of behaviours including diet and physical activity, and current smoking practices. *Maternal oral self-care*: Mothers will be asked about their own oral health and use of dental services (usually and during pregnancy), exposure to fluoride, and their history of dental treatment (fillings/extractions for caries when young). *Maternal oral health knowledge: *Value of oral health behaviours, role of services and service availability, oral health education, maternal oral health behaviours (risk and protective including frequency of brushing of own teeth and recent preventive dental health checks) and attitude variables will also be collected. *Parental support of infant oral hygiene: *items will assess the age of first brushing and frequency of brushing; items reflecting assistance with brushing, use of fluoride toothpaste, use of fluoride drops or sources other than water or toothpaste. *Feeding practices: *Nutrition behaviours and consumption patterns, feeding habits and infant nutrition (formulas and breast milk) will be assessed. Breastfeeding items will capture current and past breastfeeding habits (including prolonged breastfeeding during sleep), use of dummies/pacifiers, other drinks, introduction of solids, being put to sleep with a bottle other than water, and source of drinking water.

#### Distal variables

*Maternal health currently and in pregnancy: *items will include global health rating, illnesses during pregnancy, and medication usage. *Birth factors *(from parent-report and parent-held child health record): items for date of birth, gender, birth order, birth weight, gestational age. *Child health: *items will include a global rating of general health, chronic conditions, hospitalisations and accidents. *Current maternal mental health, stress and coping *will be assessed using the K6 Screener for psychological distress, presence of depression and perceived stress and coping. *Family socio-economic and demographic variables will include*: items on ethnicity, cultural background, maternal age, parental education, and family structure. Socio-economic status (SES) position will be assessed by the education (highest level achieved), most recent occupation (white collar, sales and clerical, blue collar, never in the paid workforce), household income (divided into quintiles), and smoking status (current smoker, past smoker, non-smoker), marital status and the family affluence scale [27]. *Residential socio-economic variables*: items assessing frequency of residential moves, number of bedrooms (as a measure of overcrowding) and residential location. Location will be geographically-coded to derive the SEIFA (Socio-Economic Indices for Area) index which provides a measure of social disadvantage, based on population attributes within ABS Census Collectors' Districts [28].

### Oral health assessment

The oral health assessment will be conducted in accordance with the International Caries Detection and Assessment System (ICDAS) II detection codes for coronal caries [29,30] (see below for further details). Examiners will be oral health practitioners who have been trained, calibrated and are familiar with the screening methodology prior to clinical data collection. Oral health assessments will be conducted on the infants at the ages of 1, 6, 12 and 18 months and on the primary care giver at the 6 month visit only. A medical history form will be completed for both the child and primary care giver prior to the assessment.

#### Oral Health Assessment

*Babies and children*: clinicians will be seated behind the study participants who will be examined lying on their mother's lap, with their head on the examiner's lap using a headlamp. *Adults*: Primary care givers will be examined sitting up in a chair and, if possible, the examiner will be positioned behind the study participant.

Teeth (where present) will be examined in order by quadrant (upper right, upper left, lower left, lower right) and surface (distal, occlusal, mesial, buccal and lingual). The buccal and lingual surfaces of each sextant (55-54; 53-63; 64-65; 75-74; 73-83; 84-85 and similarly for the permanent dentition e.g. 18-14, 13-23, etc) will be examined and each tooth surface recorded according to the ICDAS II diagnostic criteria. Oral cleanliness (debris) will be assessed in children only by visual inspection (and confirmation by wiping with gauze if needed) for plaque deposits (see table 1 for coding). For adults a blunt-ended probe may be used to conduct the Modified Sulcus Bleeding Index [31] (see below) or to confirm assessment of the tooth surface (tooth 16, 21, 24, 44, 41 and 36) using ICDASII.

**Table 1:** Classification and codes used for the clinical oral health assessments.

Assessment	Code	Descriptor
**1. Debris index^1^**	0	Absence of plaque/debris
	1	Plaque/debris visible
**2. Modified sulcus bleeding index^2^**	0	Absence of bleeding (*Healthy appearance)*
	1	Presence of bleeding
**3. Modified ICDAS II^3^**		
Teeth missing	96	Surface cannot be examined; surface excluded
		*When the examiner is unable to form a judgement of the status of the surface*.
	97	Missing due to caries
		*Part of the tooth surface has been extracted because it was carious*.
	98	Missing other than caries
		*Surfaces are regarded as extracted for orthodontic reasons, unless there is overwhelming evidence to the contrary, missing first molars will be recorded as extracted due to caries*.
	99	Unerupted teeth
		*Deciduous or permanent teeth which have not yet erupted*.
Tooth surface	0	Sound tooth surface - no evidence of caries
		*Surfaces with developmental defects are to be recorded as sound: enamel hyperplasia; fluorosis; tooth wear (attrition, abrasion and erosion); extrinsic or intrinsic stains. Surfaces with multiple stained fissures, if the condition is seen in other pits and fissures, should be scored as a sound surface*.
	1	First visual change in enamel
		*Seen only after drying or restricted to within the confines of a pit or fissure. After drying white or brown lesion is visible that is not consistent with the clinical appearance of sound enamel*.
	2	Distinct visual change in enamel
		*When wet there is: *1) Carious opacity and/or; 2) Brown carious discoloration which is wider than the natural fissure/fossa that is not consistent with the clinical appearance of sound enamel (note: the lesion must still be visible when dry).
	3	Localised enamel breakdown
		*Secondary to caries with no visible dentine or underlying shadow*. There is clear carious opacity and/or brown carious discolouration; or carious loss of tooth structure at the entrance to/within the pit/fossa/fissure when dry.
	4	Underlying dark shadow from dentin
		*Lesion appears as a shadow of discoloured dentine visible through an apparently intact enamel surface which may or may not show signs of localised breakdown*.
	5	Distinct cavity with visible dentin
		*Cavitation in opaque or discoloured enamel exposing the dentine beneath. Once dried, there is visual evidence of demineralisation and/or loss of tooth structure at the entrance to or within the pit or fissure - frank cavitation*.
	6	Extensive distinct cavity with visible dentin
		*Obvious loss of tooth structure, the cavity is both deep and wide and dentine is clearly visible on the walls and at the base. An extensive cavity involves at least half of a tooth surface or possibly reaching the pulp*.
Caries associated with restoration and sealants	0	Sound; *Surface not restored or sealed*
	1	Sealant, partial
	2	Sealant, full
	3	Tooth coloured restoration
	4	Amalgam restorations
	5	Stainless steel crown
	6	Porcelain or gold or PFM crown or veneer
	7	Lost or broken restoration
	8	Temporary restoration
	9	Used for missing teeth (see above).

^1^Children only; ^2^Adults only; ^3^ICDAS: International Caries Detection and Assessment System

#### Modified Sulcus Bleeding Index: adults only

The CPI probe tip will be inserted gently in the sulcus/crevice in a gentle sweeping motion from one interproximal space to the other interproximal space on the same tooth. After 30 seconds the tooth status will be coded according to one of the following criteria (modified from Mühlemann [31]): Absence of bleeding/Healthy appearance or Presence of bleeding (as in Table 1). Buccal and lingual surfaces of each tooth are assessed. Specific index teeth known as Ramfjord teeth can be used for a partial-mouth assessment to reduce participant and researcher physical, time and cost burden [32,33]. These are the maxillary right and mandibular left first molars, maxillary left and mandibular right first pre-molars, and maxillary left and mandibular right central incisors [34]. For the bleeding index, the Ramfjord teeth will be assessed and where a Ramfjord tooth is missing, the neighbouring tooth of the same type (molar, premolar or incisor) will be used.

For all aspects of the clinical and saliva data collection, appropriate cross-infection procedures will be in place (i.e. new pair of gloves for each subject and the use of hand disinfectant). A standardised recording form will be used to record the data. The *Federation Dentaire International *(FDI) 2-digit tooth numbering system (i.e.: 55, 54, etc) will be employed. The aim is to record the present status of teeth in terms of disease and treatment history. Where visibility is obscured, debris or moisture will be removed with gauze (after tooth cleanliness is assessed) prior to examination. Compressed air will not be used and radiographs will not be taken.

#### Indices

Each tooth surface will be coded and all participants (child and mother) will have a dental record card. Observations will be recorded according to the ICDAS II coding system (see Table 1) and will include teeth count, early signs of changes in enamel, white spot lesions and the number of cavitated lesions (decayed), missing and filled surfaces [29,30]. *White spot lesions*. White spot lesions can be viewed as evidence of early stage of the carious disease process. Diagnostic criteria developed for use in epidemiological studies enable visual differentiation between non-cavitated white spot lesions and developmental enamel defects (hypoplasia), according to the texture (matt and rough surface for the early carious lesion) and site of the lesion on the tooth surface. White spots related to pits and fissures or the contour of the gingival margin are defined as early onset carious lesions, whereas all other white spots on other tooth sites are considered as hypoplasia. Examiners for this study will be trained and calibrated for use of the codes against a 'gold standard' examiner.

#### Saliva sampling and microbiological testing

Saliva (1-5 mL) will be collected from mothers and infants. For infants a pipette will be used to extract saliva from the oral cavity. The sample will be placed immediately in sealed tubes, frozen (-20°C), stored and periodically transported to a laboratory for longer term storage at -80°C. *Detection of oral bacterial species in saliva using real time-polymerase chain reaction (PCR)*. The relative proportions of five bacterial species in saliva of infants and caregivers will be determined at each time point using standard DNA extraction and Real Time PCR techniques. Comparison of the relative proportions of each bacterial species at each time point will enable determination of a bacterial profile associated with the onset of ECC. *Fluoride determination of saliva: *Fluoride concentration in saliva will be determined from salivary samples obtained for microbial analysis, and determined after acid treatment using a fluoride specific electrode on a LabCHEM mv/pH meter, using the Vogel method [35]. *Buffering capacity of saliva: *Buffering capacity of saliva will be determined from the samples obtained for microbial analysis using a pH Stat system (Radiometer). *Calcium and phosphate analysis of saliva: *Inorganic phosphate levels will be determined by the colorimetric method of Itaya and Ui[36]

### Analysis

The presence of caries is to be established at tooth surface level and its incidence is to be monitored at this level, hence it is appropriate to perform and report primary analyses with tooth surface as the unit of analysis. Analyses will account for clustering of surfaces within children's mouths, repeated measurements over time, and the fact that most other data are collected at the individual-level of measurement. However, to permit comparisons with other published work, secondary analyses will also define ECC as individual-level child outcomes [eg. Modified ICDAS II, mean dmft, intensity of ECC index (ratio of affected teeth to erupted teeth)]. Descriptive statistics will be produced for all cross sectional outcomes (means, standard deviations, prevalence etc) and to examine distributional characteristics to inform scaling and choice of analytical techniques. Categorisation of the outcome will be considered based on clinical understanding of appropriate cut points. For exploratory analyses of relationships between ECC and biological, behavioural and environmental factors, we will employ a range of multivariable and multi-level models depending on the level of definition of ECC outcome. At the individual level, multiple linear regression and mixed models will be used for continuous outcomes; and multinomial logistic regression for categorised ECC outcomes. To examine the levels and rates of change in oral health status from ages 6 to18 months at surface level, multilevel random coefficient models and generalized estimating equations (GEEs) adjusting for clustering will be used. These techniques also permit inclusion of partial data from infants that are lost-to-follow-up.

### Ethics

Ethics approval to conduct this study has been provided by the University of Melbourne Human Research Ethics Committee (HREC 0722543) and the Victorian Department of Education and Early Childhood Development. All participants will provide informed consent before baseline data is collected.

## Discussion

To date, investigations of the development of early childhood caries have almost exclusively focussed on either biological factors or socio-environmental factors, and studies that combine measurement of both areas are rare. From a socio-ecological perspective, parent and child oral health behaviours occur within a cultural and economic context, the effects of which are mediated by individual capacities [37]. Common factors influencing child health and wellbeing include gender, co-morbid health conditions, parental physical and mental health, and the socio-economic resources of the family. Few studies have explored these issues for children in the very early years of life. The VicGeneration study will strengthen the evidence base by exploring the interactions between biological and socio-ecological factors for child oral health and providing accurate measurement of dental decay in young children at high risk. This evidence is critical to inform community-based interventions, current and future health promotion activities and public health policies, and thereby to improve child oral health across the population.

The strengths of this study include the innovative application of a socio-ecological approach; collection of longitudinal data that will enable inferences to be made about causal pathways; and the focus on a high risk sample (young children from disadvantaged, rural and culturally and linguistically diverse families) for whom preventive intervention is a public health priority.

## Abbreviations

CALD: Culturally and Linguistically Diverse; ECC: Early Childhood Caries; GEE: Generalised estimating equations; LSAC: Longitudinal Study of Australian Children; LGA: Local Government Area; MCH: Maternal and Child Health; MCHN: Maternal and Child Health Nurse; PCR: Polymerase chain reaction; SEIFA: Socioeconomic Index For Areas.

## Competing interests

The authors declare that they have no competing interests.

## Authors' contributions

EW conceived of the study, and all authors participated in its design and methodological development. AdS drafted the initial manuscript with assistance from all authors, who also read and approved the final manuscript.

## Pre-publication history

The pre-publication history for this paper can be accessed here:

http://www.biomedcentral.com/1471-2458/10/97/prepub
